# Immersive experiences in museums for elderly with cognitive disorders: a user-centered design approach

**DOI:** 10.1038/s41598-024-51929-4

**Published:** 2024-01-23

**Authors:** Xinyue Yi, Zhizheng Liu, Hong Li, Bo Jiang

**Affiliations:** 1 School of Architecture and Art, Guangxi Arts University, Nanning, 530009 China; 2 School of Design and Art, Beijing Institute of Technology, Beijing, 102488 China; 3 Faculty of Innovation and Design, City University of Macau, Macau, 999078 China; 4 Guangzhou Huashang College, Guangzhou, 511300 China

**Keywords:** Psychology, Mathematics and computing

## Abstract

In the context of global aging, to explore the design needs of elderly with dementia in museum environments, to establish a user cognitive psychological model based on immersion theory, and to enhance the satisfaction of cognitively impaired dementia with the museum service experience. Using literature research, surveys, questionnaires, interviews, and focus groups, we analyze the experience design from the psychological demands of elderly with dementia, build a method of mining user needs by combining the KANO model with the analytic hierarchy process (AHP) method, and establish a model for evaluating the excellence of the experience of the museum environment. The conclusion shows that displaying museum virtual scenes or old objects can effectively increase the subjective well-being of people suffering from various health conditions. The method can accurately tap the attributes of the needs of elderly with dementia, break through the drawbacks of the traditional museum experience design which is dominated by the designer's subjective consciousness, and allow the audience to better experience the museum immersive experience, which provides a new idea and method for the effectiveness of cognitive interventions for elderly with cognitive disorders.

## Introduction

According to the World Health Organization's Global Status Report on Responding to Dementia in Public Health, more than 55.2 million people worldwide are suffering from dementia in old age in 2019. Alzheimer's disease is the most common form of dementia and has a prevalence of about 8.1% for women and 5.4% for men among those 65 and older. There will likely be 78 million dementia sufferers worldwide by 2030^1^. China has a vast population, making it one of the nations with the fastest increases in the global dementia population. In 2020, Longfei Jia et al. concluded that there were 15.07 million people in China aged 60 and older with dementia and another 38.77 million with mild cognitive impairment (MCI), according to a study looking into the prevalence, risk factors, and management of dementia and mild cognitive impairment^2^. Overall, there is a huge gap in China's understanding of dementia and screening for it, as well as problems with dementia care and treatment^3^.

Pre-clinical, mild cognitive impairment, mild dementia, moderate dementia, and severe dementia are the five phases of dementia. The ideal phases of the disease to treat are the preclinical and mild stages, however, due to inadequate scientific publicity, public awareness of and understanding of dementia in old age is limited and only on a superficial level. The drugs currently approved for dementia in older adults are mostly used to treat the complications caused by dementia, and only help to improve the quality of survival for elderly with cognitive disorders, with no obvious therapeutic effect on the disease itself, and are prone to adverse side effects and even increase the risk of death for elderly with cognitive disorders, according to the academic studies that are currently available^4^. More researchers are advocating focusing on non-pharmacological treatments, such as active environmental therapy^5^, life care, vocational training, cognitive rehabilitation therapy, creative storytelling^6^, music therapy, and pet therapy^7–10^, given the generally low level of medication adherence. As a result, the trend towards non-pharmacological treatment for dementia in older adults involves the adoption of contemporary techniques to develop cutting-edge solutions to reduce symptoms of elderly with cognitive disorders.

### Museums and cognitive interventions

Modern museums have created several cutting-edge techniques to promote health and well-being as a result of the growth of digital technology, utilizing the power of spatial surroundings to help people feel less alone, relieve pain, elevate their moods, and improve their memories.

Theoretically, researchers have looked at museums and elderly with cognitive disorders health from a variety of angles. For instance, in 2013^11^, Helen Chatterjee and Guy Noble publicly developed the idea of "museums in health," which investigates, evaluates, and justifies the usefulness of museums in terms of fundamental physiological and psychological principles. The importance of museums to the general well-being of the country is examined in terms of fundamental physiological and psychological principles. The good impacts of museums on the physical and mental health of older people, persons with illnesses (such as Alzheimer's disease and stroke), and other groups have been confirmed by a 2014 study by the Research Centre of Museums and Galleries (RCMG) at the University of Leicester in the UK^12^.

On a more practical level, some academics have suggested that museums should offer services to assist disadvantaged groups in society, enhance facilities for disadvantaged groups that provide humane services, create thematic activities related to disadvantaged groups, plan the content of exhibitions^16^, and travel to these groups' ghettos^13–15^. To expand their social engagement and influence and to help underprivileged groups develop their social skills and integrate into society, museums in Europe and the United States have created educational programs^17^. For instance, the MoMA Museum of Modern Art in New York has started a program called "Meet Me at MoMA" for those who have dementia. The program invites those who have mild dementia and their family or carers to the museum to visit the exhibition and engage in conversation about the work. The initial intention is to increase awareness of elderly with dementia and encourage integration between elderly with cognitive disorders and their carers through watching and discussing the art. The Museum of Old Things in Rotterdam, the Netherlands, houses a collection of commonplace items that have been gathered from all over the nation and arranged in different thematic areas like the living room, kitchen, bedroom, and tool room. These familiar items help elderly people with dementia feel less lonely and have a significant therapeutic effect on memory. For research on creative narrative therapy, the aforementioned study on the current state of museums for the treatment of elderly with dementia serves as a point of reference^18^.

### Creative storytelling therapy and immersion theory

Cognitive therapies are frequently utilized in the early and middle stages of dementia^19^, with creative storytelling therapy being the more popular method^20^. Dr. Basting who thought that everyone, even those with dementia, is creative and that creativity develops new ideas and provides something of value to the world in new ways^21^, developed creative storytelling for older adults with dementia in 1996. The goal of creative storytelling is to improve older people with dementia's communication and expression skills, their relationship with their carers, and their quality of life^22^. Social workers use pictures and multimedia to stimulate older people with dementia's creativity and imagination through open-ended questioning, collaboration, and sharing. According to Epstein's dual process hypothesis, which was put forth in 1994^23^, humans process events using a combination of cognition and emotion. The abundance of objects that jog people's memories is clear to see as the key to the creative storytelling process. Museums, as a means of "collecting memories," have a key resource at their disposal as well as a key entry point for museums to offer reminiscence therapy services to the dementia community^24^.

In their study, Kim et al.^25^ and Coyle et al.^26^ shown that VR therapies can positively affect a range of clinical outcomes in elderly with cognitive disorders with cognitive impairment and that they can also only slightly enhance cognitive functioning in those participants. When older people with dementia focus all of their effort on imaginative storytelling, they experience a high level of excitement and enrichment, which promoting cognitive sense enhancement in cognitively impaired older adults^27^. Virtual reality environments can help create safe memory environments for people with dementia. Contrarily, immersion is at the core of virtual reality (VR) technology, which refers to the use of computer-generated, three-dimensional virtual environments. In these environments, users interact with virtual objects using sensory devices that make them feel as though they are in the real world, experiencing a sense of immersion in the virtual world's scenario. The emergence of immersion is inextricably linked to a high level of engagement, and emerging media like virtual reality, augmented reality, and mixed reality can produce intensely interactive experiences that allow space to significantly contribute to an incredibly strong sensation of immersion^28–30^.

The prominent American psychologist Mihaly created the immersion hypothesis, also known as the mind-flow experience, in 1975 to explain the psychological experience of persons who are intensely focused on a task and reach a state of unconsciousness as a result^31^. Visitors who enjoy an immersive experience are better able to focus on the environment and leave with a more in-depth cognitive understanding of what is on display there. With improvements in science and technology, VR is being used more frequently and is starting to enter the medical industry^32^ for the treatment of a variety of elderly with cognitive disorders. Through the use of para-naturalistic, realistic stimuli during experiencing services, people with lower cognitive abilities can be implemented in a multimodal way^33–35^. Therefore, the use of immersion theory based on VR technology in museums and creative storytelling-assisted therapy for elderly with cognitive disorders can significantly improve visitors' visual experiences in museums and, with the introduction of tactile gloves, further improve elderly with cognitive impairments' tactile experiences in the virtual world. Reminiscent stories can be written to create a fully immersive experience that supports cognitive and emotional functioning and induces a state of mental and physical immersion^36^.

### Research questions

It is clear that over the past ten or so years, the public has gradually come to accept creative narrative therapy, a non-pharmacological therapeutic method used in museums, as a result of the advancement and popularity of technology and knowledge. The current research on creative narrative therapy has some limitations, not the least of which is the paucity of studies on the design of cognitive illness museum space experiences based on immersion theory. Additionally, the disparity between experience service design and audience cognition, a lack of attention to the situation of scene creation management function, and other factors have increased the variability of experience and services in different museums, which has a direct impact on the effect and satisfaction of visiting experiences of elderly with cognitive disorders. Last but not least, it is difficult to directly apply the demand excavation in the development of recollective therapy in museums because it is frequently based on the designer's subjective design consciousness. Therefore, rather than focusing on a single factor that might be cognitively biased, this study needs to take into account the practical requirements of elderly with cognitive impairment and the combined impact on immersion states in terms of design implications in terms of museum service experiences.

For elderly with cognitive disorders, this study suggests combining creative narrative therapy with immersion theory to improve the effectiveness of service experiences. A set of service experience design strategies for elderly with dementia in museum scenarios is established to improve the immersion of cognitively impaired people during cognitive rehabilitation training to maintain the intervention by using the KANO model and the analysis method of AHP to more precisely explore the attributes of needs of elderly with cognitive disorders. The objective is to increase persons with cognitive impairments' involvement in cognitive rehabilitation training, to retain the impact of interventions to boost the effectiveness of rehabilitation and to offer design guidelines for future museum service experiences.

This study addresses the following key questions to help designers better understand and capture the changing needs of people with dementia:

1. Identifying which design features better reflect the service demands of elderly with cognitive disorders and enable better immersion is essential for identifying needs of elderly with dementia.
2. More research on dementia’s preferences for features of multi-level designs that are motivated by their needs.
3. Use the research's findings to draw inferences about the variables that affect crucial choices in the creation of museum service experiences.

## Research methodology

### KANO-AHP research methodology

This subsection will briefly introduce some basics of the KANO model and the analytic hierarchy process (AHP) method.

#### KANO model

**Figure 1:**
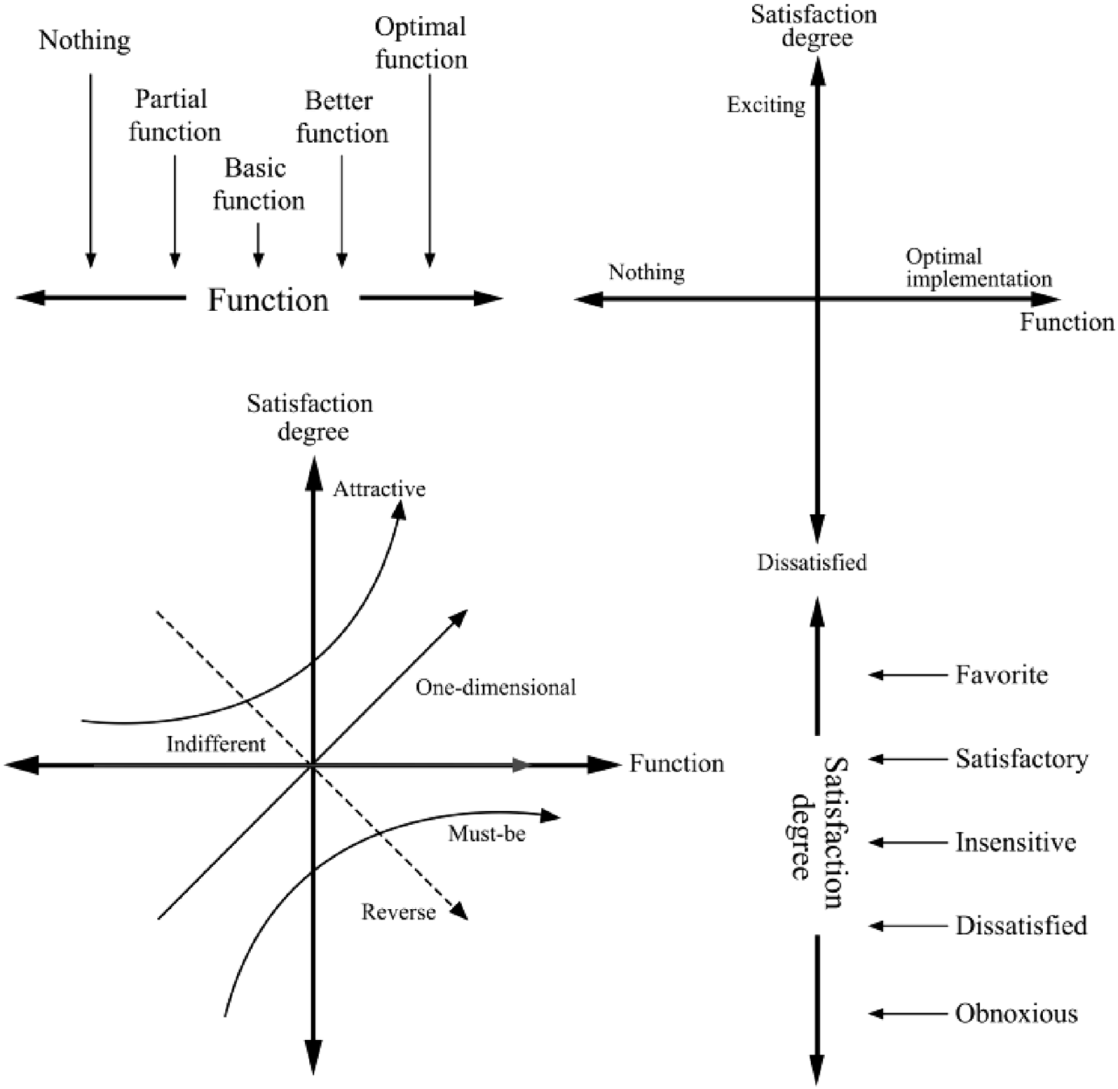
KANO model element relationship.

The KANO model, a conceptual model proposed by Noriaki Kano, a professor at Tokyo Institute of Technology, is a research method for evaluating audience needs and effectively classifying the attributes of the needs in the face of the design research related to audience needs^37^. In this study, the KANO model was used to accurately classify the demand attributes of dementia by focusing on their needs, which can be used to tap into the emotions and perceptions of cognitively impaired people's different needs for spatial experiences through a nonlinear approach, and can specifically classify audience needs into the following attribute types: Must-be attribute, One-dimensional attribute, Attractive attribute, Indifferent attribute, and Reverse attribute, as shown in Fig. 1.

#### AHP method

The Analytical Hierarchy Process (AHP) was introduced by Scary in 1971 as a method of analysis linking quantitative and qualitative^38^. The logical judgement of the AHP as a simple hierarchical weighting decision analysis method is very scientific and structured. Using this method can scientifically further dissect the complex elements derived from the preliminary KANO model, calculate the weight value of each spatial experience element, and help designers to prioritize the spatial experience design elements of cognitively friendly museums.

### Model construction based on KANO-AHP

**Figure 2:**
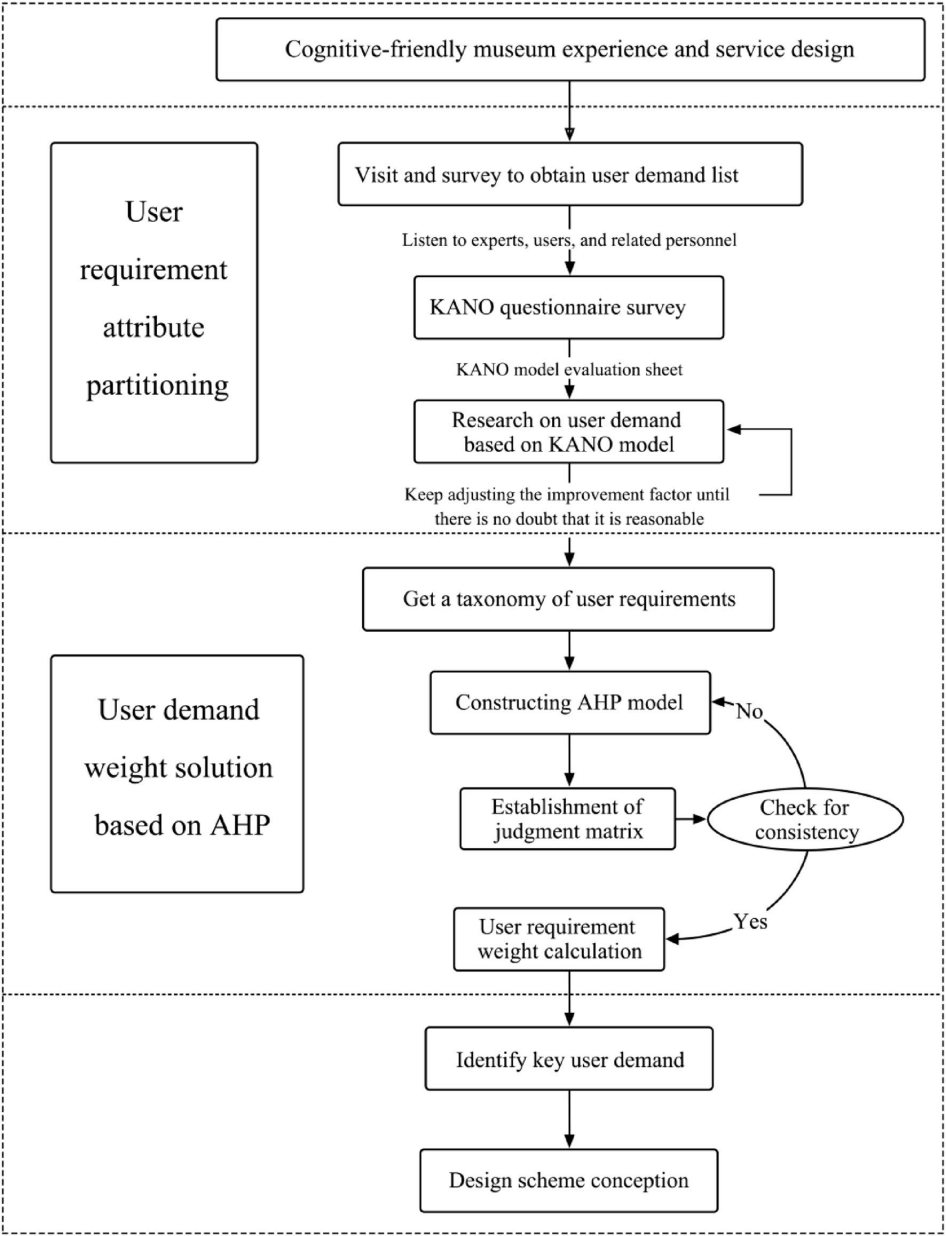
Model flow.

The primary premise for the KANO model attribute classification to arrive at the solution idea was the weighting of the needs of senior individuals with cognitive impairment in the research of museum space experience design. The traditional KANO model's weighting solution is primarily based on the Better-Worse index analysis and the Delphi method, which do not accurately reflect the importance of needs of older adults with cognitive disorders^39^. On the one hand, the KANO model has limitations in that it is difficult to intuitively reflect the hierarchy of importance in the ranking of need attributes after needs of older adults with dementia have been extracted^40^. The AHP technique has the properties of simplicity, science, and system, and can solve the weights of each index fast and accurately in complicated multi-criteria decision-making issues^41^. As a result, the AHP technique can be applied to the KANO model to perform the audience demand weighting operation, which can reflect its importance ranking. The KANO model attribute classification type may be utilized as the foundation for the qualitative hierarchical indicator model in the AHP. It also guarantees the impartiality of the audience demand hierarchy analysis model in AHP. The typical weighting operation in the KANO model is solved. The two approaches can be combined to create a museum service experience design model; its research model and framework are shown in Fig. 2.

It goes like this to provide a museum service experience that caters to the needs of those who have cognitive impairment.

1. With KANO, identifying and categorizing the characteristics of visitors with cognitive impairments' demands for spatial experiences in museums.
2. To determine the primary audience requirements for the concept, the weighting of the indicators for the various aspects of spatial experience demands of older adults with cognitive disorders was resolved using AHP.

### Classification of audience demand attributes based on the KANO model

1. Conducting research to identify and document the requirements of many individuals with cognitive impairment in the design of museum space experiences.
2. Designing questionnaires and conducting systematic research using the KANO model.

The research participants and their families were asked to complete each positive and negative question for each need, which had five alternatives, according to how each needs related to the dementia experience was built up as a questionnaire with two positive and negative questions, as shown in Table 1.

**Table 1:** KANO questionnaire.

Option		Dislike	So-so	Apathy	Like	Like best
Problem	Forward: design the requirement	1	2	3	4	5
	Reverse: the requirement is not designed	1	2	3	4	5

(3) KANO evaluation form

The KANO questionnaire survey findings were compared to the KANO evaluation table (as shown in Table 2), and the needs were divided into four categories: must-have, one-dimensional, attractive, non-different, and reverse qualities. The goods on demand for the two categories of qualities were disregarded.

**Table 2:** KANO evaluation table.

User demand		Reverse problem				
		Dislike	So-so	Apathy	Like	Like best
Forward problem	Dislike	Q	R	R	R	R
	So-so	M	I	I	I	R
	Apathy	M	I	I	I	R
	Like	M	I	I	I	R
	Like best	O	A	A	A	Q

*M* must-be attribute, *O* one-dimensional attribute, *A* attractive attribute, *I* indifferent attribute, *R* reverse attribute, *Q* questioned result.

### AHP-based solution for experience demand weights

**Figure 3:**
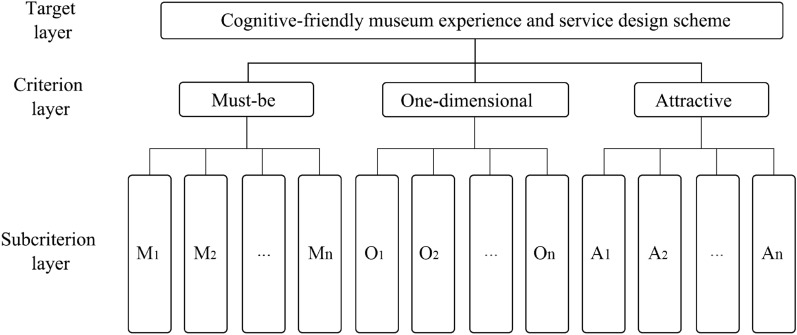
Analytic hierarchy process model based on KANO.

The AHP technique calculates the importance of each audience's need when designing museum experience services. The museum space design scheme is primarily set as the target layer, the criterion layer is a must-be attribute, a one-dimensional attribute, and the AHP model was established by using the demand items unmet (as shown in Fig. 3). Next, according to the dominant relationship of the hierarchical analysis method and the principles of hierarchical analysis model construction, combined with the KANO model demand attribute division type. Furthermore, to resolve the demand weights of each layer and make clear the ranking of the significance of each demand, a suitable judgment matrix was built and a geometric mean algorithm^42^ was introduced. The audience needs that should be addressed in the museum design were then determined based on the ranking of the relevance of demand of older adults with cognitive disorders. The audience demands that should be satisfied in the museum design were then determined based on the ranking of the priority of wants of older adults with dementia to provide conceptual direction for the ensuing experience service design scheme.

### Kano model analysis

This study's focus is on the design features of a museum experience service for people with cognitive impairment. This study is separated into four process steps in the Kano model research phase to scientifically tap into the features of fundamental needs and other influencing elements that may be taken into account while creating.

Phase 1 of the data collection process. For several audience research projects, stakeholders' demands were assessed using observation, interviews, and questionnaires. Initially, a sample of design elements from the most popular medical science websites—Wikipedia, sciencedaily.com, Penguin Medical Dictionary Public, World Health Organization, National Center for Disease Control, Dingxiang Doctor Public, guokr.com, haodf.com, etc.—was gathered; next, by logging into each of these websites, the Alzheimer's disease-related experiential service features were accessed and recorded; finally, from the perspective of older adults with cognitive disorders, the features were recorded. The researcher combined the aforementioned sample with five focus group interviews (5 participants), ten unstructured interviews with people with cognitive impairment (as well as family members), museum design and planning experts, and academic experts after conducting a review of the pertinent literature. The findings of the interview research were compiled to produce 25 expected need items based on comments and discussions from the interviews, which produced the original data on the requirements of people with cognitive impairment.

The next stage of the survey study phase. A questionnaire star internet survey was used to distribute the questionnaire to 280 participants. The questionnaire was intended for four main target audiences: professionals and academics in the field of cognitive impairment research, psychologists working in museums (industrial design and psychology, technology use), people with mild to moderate cognitive impairment (and their carers), and general visitors. See Table 3 for a sampling of the survey's demographics. The audience questionnaire's original demands were graded on a 5-point Likert scale with scores of very important, very important, average, unimportant, and extremely unimportant to initially weed out unimportant audience experience needs.

**Table 3:** Sample demographics for the survey.

Category	Specialist status	Age	Qualifications	Crowd share (%)
Academic experts	Professor	50–60	Doctoral degree	5
	Associate Professor	41–49	Doctoral degree	10
	PhD student or researcher	28–38	Master's degree	5
Museum Design Specialist	Health and Safety Manager	35–45	Masters/PhD	5
	Design development staff	35–45	Masters/PhD	5
	Product technicians	28–35	Bachelors/Postgraduate degrees	5
People with dementia and caregivers	Mild to severe cognitive impairment	58–69	/	20
	Caregivers	30–45	/	10
	Their family members	20–60	/	20
Public	General Visitors	22–50	/	15

Since the questionnaire research phase involves people with mild cognitive impairment and neuropsychological tests are valuable in quantifying diagnosis, the Montreal Cognitive Assessment (MoCA) scale, the most generalised scale, was used in this study as an inclusion criterion for researching cognitively impaired people. The MoCA scale is a 30-point, 11-item test of overall cognitive functioning (including short-term memory, delayed recall, visuospatial ability, verbal fluency, attention, orientation, etc.) administered over 10 min, with higher scores being associated with better cognitive functioning^43^. The classification of MoCA cognitive functioning results is as follows: 26 points for normal, 26 points for 18–26 mild cognitive impairment (MCI), 10–17 moderate, and less than 10 severe. In view of this, people with scores in the 11–25 range were selected as the main cognitively impaired people for the study.

The third level of data analysis. 272 valid questionnaires in all were gathered. By combining the research findings, the responses of each respondent were examined by the Kano evaluation form to establish the Kano category of each demand. Next, the relative majority principle was used to establish the Kano category of each demand item for all users, and finally, 20 initial demands were optimally screened, as shown in Table 4.

**Table 4:** Expected audience needs for the museum experience.

No	Demand items	No	Demand items
1	Virtual scenes	11	Music recovery
2	Situational cognitive	12	Multi-sensory interaction
3	Dementia science	13	Audio-visual presentations
4	Nostalgia therapy	14	Social context service
5	Recollections of narrative scenes	15	First aid station
6	Therapeutic exercise service	16	Health emergency equipment
7	Nursing knowledge	17	Emotional cognitive training
8	Knowledge games	18	Pet memories
9	Disease science demonstration	19	Cognitive feedback service
10	Memories postcard	20	Personalized experience

The fourth phase involves using the Kano model. The KANO model process was used to categorize the above 20 audience needs into attributes after compiling the audience research. A KANO research questionnaire was then created based on the audience need items, and it included positive and negative questions for each audience need to help determine the type to which it initially belongs, as shown in Table 5.

**Table 5:** KANO questionnaire.

Demand term	Dislike	So-so	Apathy	Like	Like best
Provide intelligent display requirements	1	2	3	4	5
Does not provide intelligent display requirements	1	2	3	4	5

According to the data of this research, the reliability of the questionnaire was analyzed by SPSS 23.0 statistical software, and the value of Cronbach's alpha for the positive questions was 0.805, and the value of the negative questions was 0.812, which indicates that the research questionnaire has a good internal consistency, and the results of the survey have credibility. The results of the statistical analysis based on the KANO assessment form for each demand item were used to more precisely determine the audience's wants, and the findings are displayed in Table 6.

**Table 6:** Analysis of results.

Demand attributes	I	Q	A	M	R	O	Demand attributes
First aid station	148	13	28	38	8	37	I Indifferent
Audio-visual presentations	134	10	30	39	17	42	
Knowledge games	128	12	40	32	14	46	
Situational cognitive	51	16	41	108	12	44	M Must-be
Dementia science	34	11	39	148	7	33	
Health emergency equipment	34	8	38	162	12	18	
Nursing knowledge	30	8	38	157	9	30	
Disease science demonstration	41	17	32	140	4	38	
Multi-sensory interaction	40	15	37	49	14	117	O One-dimensional
Pet memories	37	6	35	31	12	151	
Cognitive feedback service	47	11	32	38	16	128	
Memories postcard	29	8	54	35	9	137	
Personalized experience	29	12	47	45	11	128	
Music recovery	35	15	37	43	8	134	
Virtual scenes	34	8	138	32	14	44	A Attractive
Nostalgia therapy	52	10	120	43	17	30	
Social context service	41	15	130	29	9	48	
Recollections of narrative scenes	45	9	126	41	14	37	
Therapeutic exercise service	32	15	148	40	8	29	
Emotional cognitive training	37	12	135	48	10	30	

As a result, the first aid stations, emergency supplies, and health knowledge games are classified as indifferent needs in Table 5. As a result, the design does not take these service elements or experience design considerations into account. Situational cognitive, dementia science, health emergency equipment, nursing knowledge, and disease science demonstration are among the must-be requirements. By designing these functions, we can influence the audience's level of satisfaction. The design will concentrate more on these qualities to increase market competitiveness because the audience's one-dimensional needs, also known as linear needs, will increase significantly in satisfaction when they are met in large numbers and vice versa. When the feature of the Attractive needs is not available, it will not affect the change in audience satisfaction, but when it is designed to provide these features Therefore, the potential spatial needs of audiences will be greatly increased if the design meets their needs for virtual scenes, nostalgia therapy, social context services, Recollections of narrative scenes, therapeutic exercise services, and emotional cognitive training. This will increase the loyalty of their audiences.

### Construction of the AHP model

**Figure 4:**
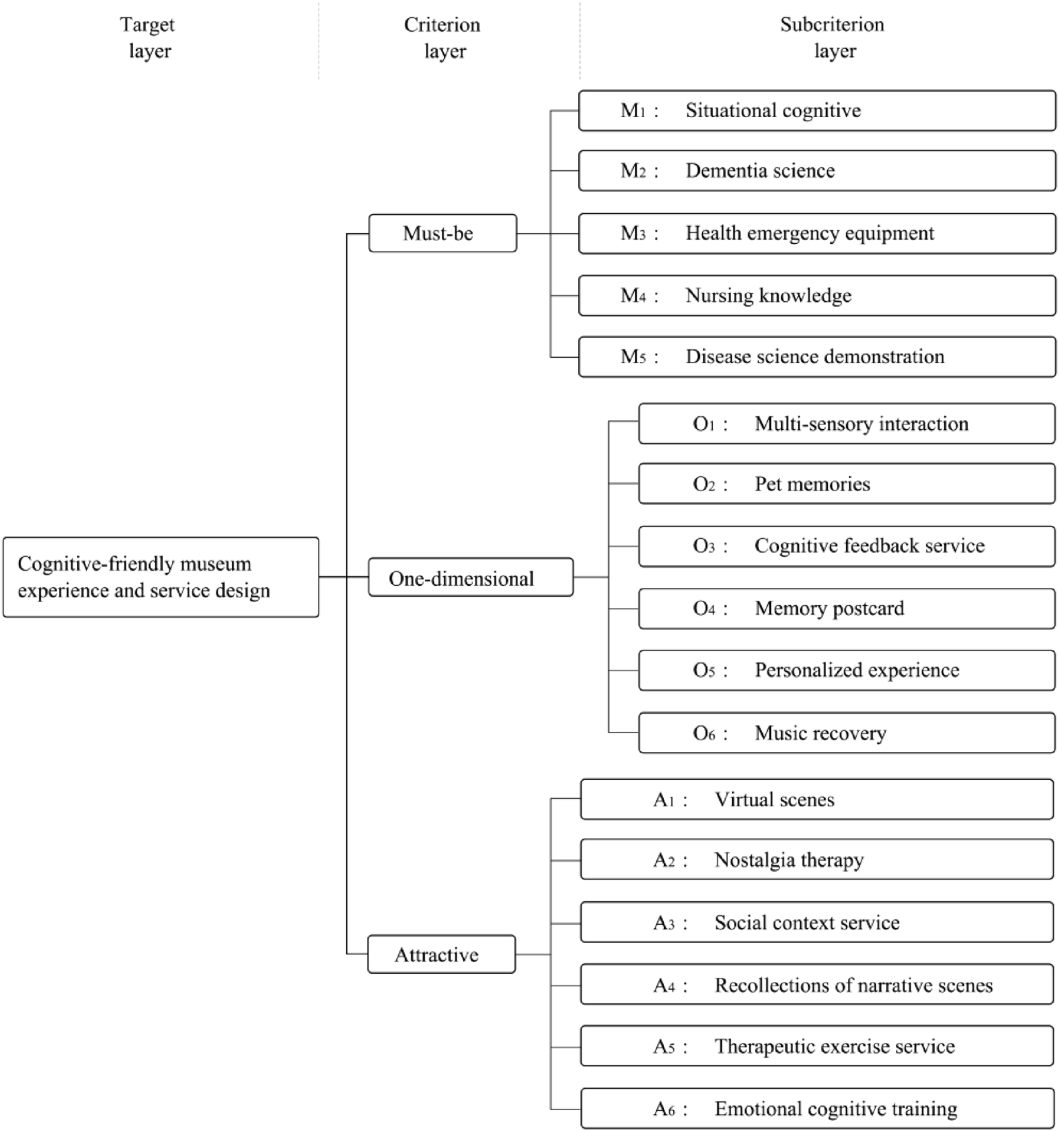
Analytic hierarchy process of cognitive friendly museum design.

The hierarchical tower ranking of the significance of the need attributes cannot be shown visually even after using the KANO model to precisely classify the audience needs. The KANO model and the AHP approach are combined to compute the weighting of each audience's needs, precisely summarizing the crucial audience needs in the design process and ensuring an objective and scientific design process. As shown in Fig. 4, the attribute division is mostly based on the findings of the KANO attribute demand analysis, and a hierarchical analysis model is built using the AHP.

To guarantee the precision of the calculation results of the service experience design needs of the cognitive disorder-friendly museum, as well as to take into account the rational and scientific weighting calculation findings. The research is conducted by using the expert opinion survey method, which, also known as the "Delphi method", refers to the way that uses a certain topic or question to solicit opinions from relevant experts or authorities. Relying on the knowledge and experience of experts has an important decision-making effect on the judgment, evaluation and prediction of research problems, and the number of experts will not be too much impact on the The accuracy of the final calculation results^37^, the method needs to go through three to four rounds of information feedback, in each feedback so that the investigation team and the expert group can carry out in-depth research, so that the final results can basically reflect the basic ideas of the experts and their knowledge of the information, and the results are more objective and credible^44^. Therefore, experts in the field, including 10 museum design-related staff, four professors in the field of dementia intervening research, three industrial designers, and three graduate students in the field of VR technology design, were asked to fill out the hierarchical analysis judgment matrix. These four groups are representative of the fields of museum design and dementia healing in old age, choosing them as research subjects can improve the accuracy and reliability of the study. The subjects were asked to rate the needs of each tier on a scale from 1 to 9 using a two-by-two comparison. The average was then used as the basis for weight calculation to derive the judgment matrix for each tier, and the geometric mean algorithm^45^ was then used to solve for the weight values of the needs of each audience in the cognitively friendly museum. The operational process is shown below, and the results of its solution are shown in Table 7.

**Table 7:** Judgement matrix and weighting values for each audience need.

Weighting hierarchy	Comparison of attributes							Weighting
Guideline level	X	O	A	M				Weighting values
	O	1	3	1/2				0.334
	A	1/3	1	1/3				0.141
	M	2	3	1				0.525
Required demand weights	M	M_1_	M_2_	M_3_	M_4_	M_5_		Weighting values
	M_1_	1	2	4	2	3		0.365
	M_2_	1/2	1	4	2	2		0.258
	M_3_	1/4	1/4	1	1/2	1/2		0.074
	M_4_	1/2	1/2	2	1	3		0.190
	M_5_	1/3	1/2	2	1/3	1		0.113
Expected demand weighting	O	O_1_	O_2_	O_3_	O_4_	O_5_	O_6_	Weighting values
	O_1_	1	2	1/3	2	2/3	2	0.160
	O_2_	1/2	1	1/4	2/3	1/3	2/3	0.074
	O_3_	3	4	1	3	2	3	0.353
	O_4_	1/2	3/2	1/3	1	1/2	3/2	0.110
	O_5_	3/2	3	1/2	2	1	2	0.206
	O_6_	1/2	3/2	1/3	2/3	1/2	1	0.096
Charisma demand weighting	A	A_1_	A_2_	A_3_	A_4_	A_5_	A_6_	Weighting values
	A_1_	1	2/3	2	1/3	4/3	2	0.143
	A_2_	3/2	1	5/2	1/2	2	3	0.208
	A_3_	1/2	1/2	1	1/5	2/3	4/3	0.080
	A_4_	3	2	4	1	4	4	0.389
	A_5_	3/4	1/2	3/2	1/4	1	3/2	0.107
	A_6_	1/2	1/3	3/4	1/4	2/3	1	0.074

(1) Solve for the product of the scales in each row.

1 $$M_{i} = \prod\limits_{j = 1}^{m} {b_{ij} } { (}i = 1,2,...,n)$$

where: $b_{ij}$ represents the audience demand indicator in row $i$ column $j$ and m represents the amount of audience demand indicator.

(2) Operation of the geometric mean of the product of the scalars of each row.

2 $$a_{i} = \sqrt[m]{{M_{i} }} \, (i = 1,2,...,n)$$

(3) Calculation of relative weights.

3 $$W_{i} = \frac{{a_{i} }}{{\sum\limits_{i = 1}^{m} {a_{i} } }}$$

(4) Solve for the largest characteristic root.

4 $$\lambda_{\max } = \frac{1}{n}\sum\limits_{i = 1}^{n} {\frac{{B_{{W_{i} }} }}{{W_{i} }}}$$

where:$B_{{W_{i} }}$ represents the $i$-th component of the vector $B_{W}$ and n represents the order.

(5) Test of consistency of results.

5 $$CI = \frac{{\lambda_{\max } - n}}{n - 1}$$

6 $$CR = \frac{CI}{{RI}}$$

where: $n$ indicates the number of orders corresponding to the evaluation scale of the judgment matrix; $RI$ represents the average random consistency index, with corresponding values for each order, as shown in Table 8; $CR$ indicates the consistency ratio, if $CR$ ≤ 0.1, it indicates that the consistency test is passed; when $CR$ > 0.1, it means that the consistency test is not passed, and the judgment matrix needs to be checked and corrected and adjusted before calculating and analyzing again.

**Table 8:** Average random consistency indicators.

n	1	2	3	4	5	6	7	8	9
RI	0	0	0.52	0.89	1.12	1.26	1.36	1.41	1.46

To ensure that the testers' thinking remained consistent during the process of filling in the judgment matrix, consistency tests were conducted on the calculated results. In the analysis of the results of the AHP research method, the smaller the CR value, the better the consistency of the judgement matrix, in general the CR value is less than 0.1, the judgement matrix meets the consistency test; if the CR value is greater than 0.1, it means that there is no consistency, and it should be adjusted appropriately after the judgement matrix is analysed again. This paper for the judgement matrix calculated CI value and RI value as shown in Table 9, calculated CR value are less than 0.1, means that the research judgement matrix to meet the consistency test, the calculation of the weights have consistency.

**Table 9:** Consistency test results.

	X	M	O	A
λ_max_	3.054	5.168	6.079	6.030
CI	0.027	0.042	0.016	0.006
RI	0.520	1.120	1.260	1.260
CR	0.052	0.038	0.012	0.005

### Ethics statement

Ethical review and approval was not required for the study on human participants in accordance with the local legislation and institutional requirements.

### Statement of approval for human experiments

Identifies the institutional and/or licensing committee that approved the experiments, including any relevant details. Confirms that all experiments were performed in accordance with relevant named guidelines and regulations. Confirms that informed consent was obtained from all participants. All of the experimental procedures involving human were con-ducted in accordance with the Institutional guidelines of Guangxi Arts University, China.

### Informed consent

Informed consent was obtained from all subjects and our legal guardian(s) involved in the research.

## Results

The following conclusions can be drawn from the audience demand weight calculation findings when used in conjunction with the KANO model operation: first off, the weight values of the critical demand attributes considerably outweigh the weight values of other attributes at the criterion level from the perspective of aiding elderly with cognitive disorders in intervening. Secondly, the demand of older adults with dementia, their families, and others for the intervening service of situational awareness occupy the majority of all the essential demand characteristics, according to the level of the essential demand attributes looking at the desired demand and the charm demand. Thirdly, the major demand for health emergency equipment, nursing expertise, disease science demonstration, and dementia research at the level of required demand attributes. Fourthly, the audience is particularly interested in the demand for cognitive feedback services. Moreover, the demand for personalized experiences and multi-sensory contact is seen as a significant demand attribute, including the want for memory postcards, music recovery, and pet memories. Last but not least, when it comes to the weighting of charm demand attributes, people are more likely to rank narrative scene recall and object recollection as more significant demand attributes, followed by virtual scenes, social context services, and emotional cognitive training requirements.

**Figure 5:**
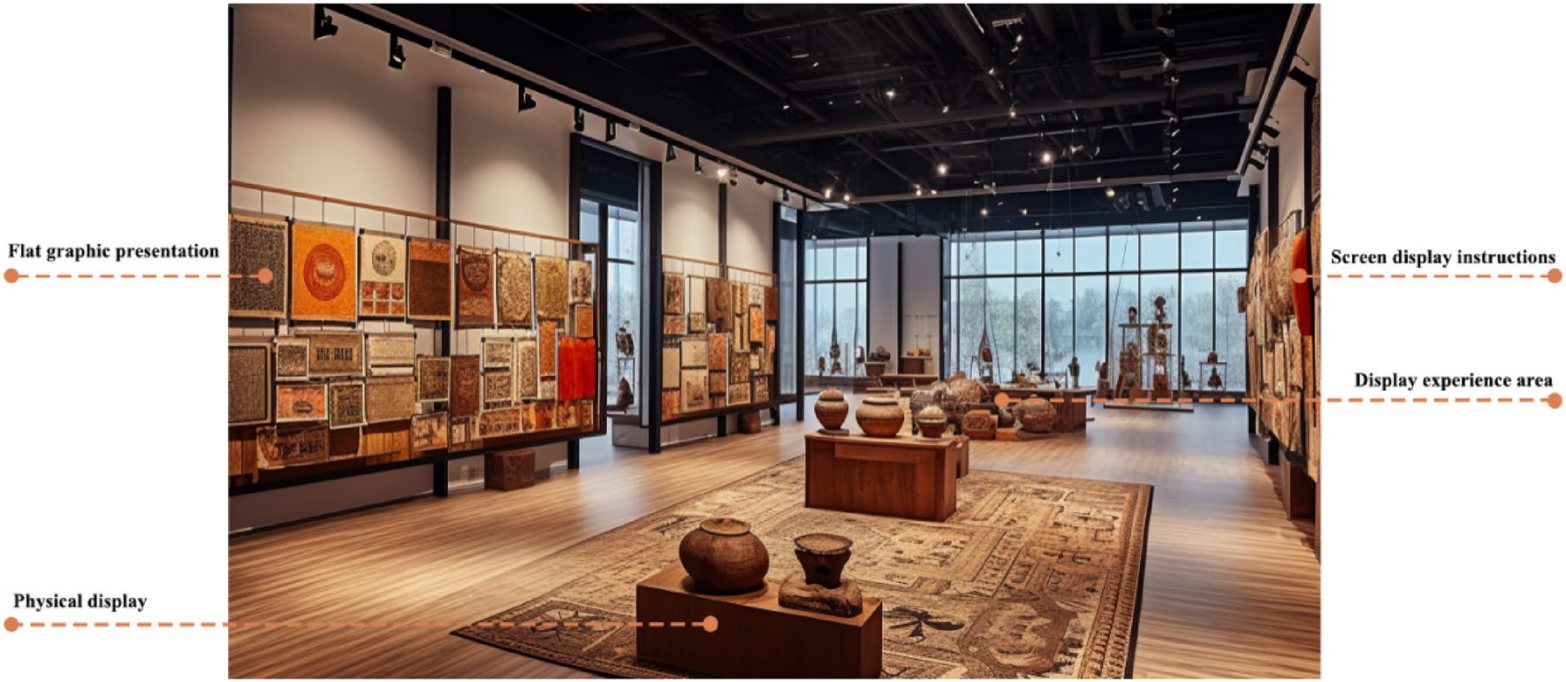
Common museum design (images are from the author's own drawings, so there are no copyright issues involved).

**Figure 6:**
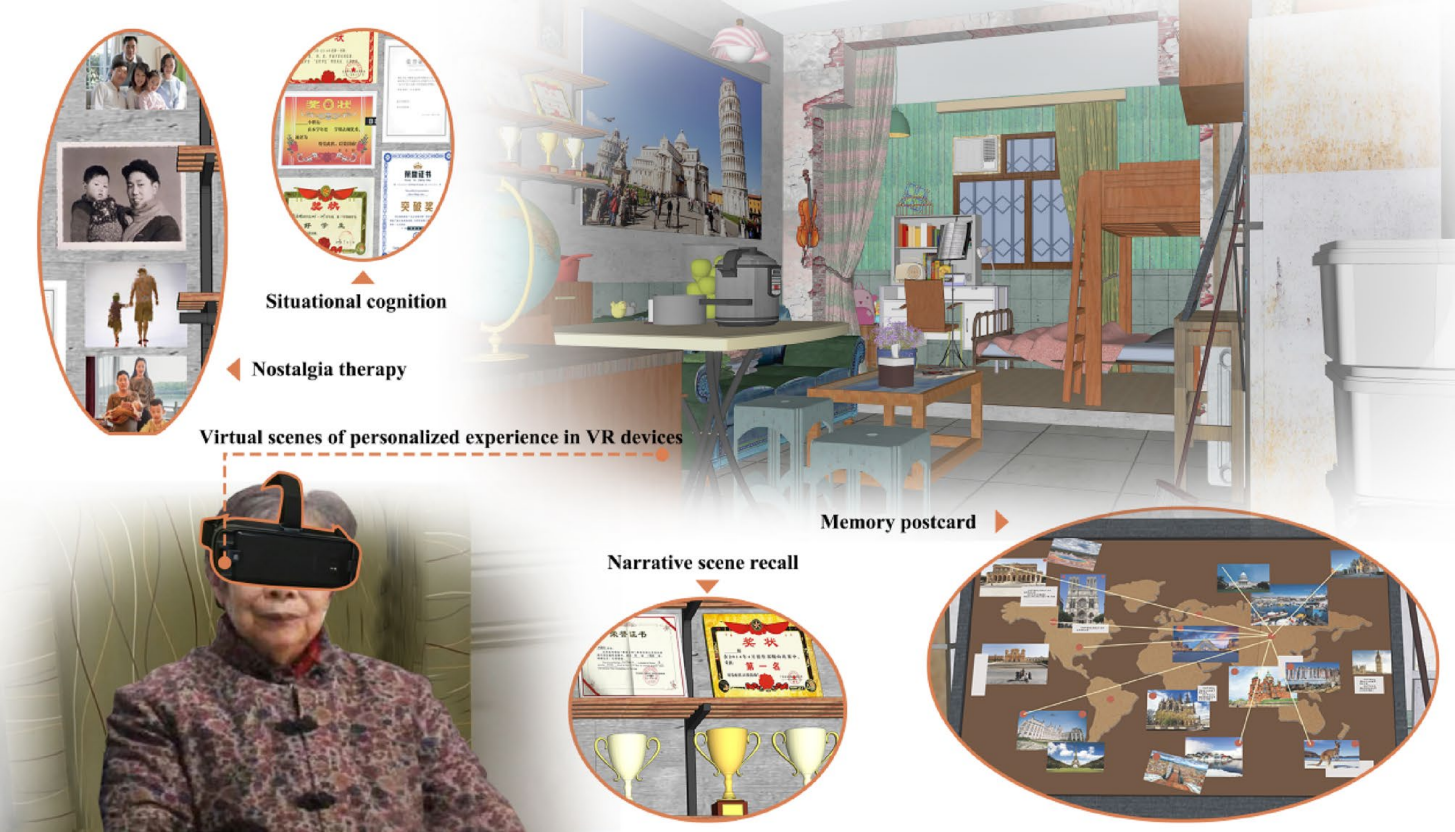
Cognitively friendly museum conceptual design scheme based on user needs (images are from the author's own drawings and permission has been obtained from the characters involved, so there are no copyright issues involved).

To assess whether the preferred cognitively friendly museum service design solution meets the audience's needs, common museum designs currently available on the market are selected for comparison with the preferred design conceptual solution. The effectiveness and feasibility of the method for cognitively friendly museum service design are verified through a comparative analysis of the original design samples and the conceptual design solutions, as shown in Figs. 5 and 6.

**Figure 7:**
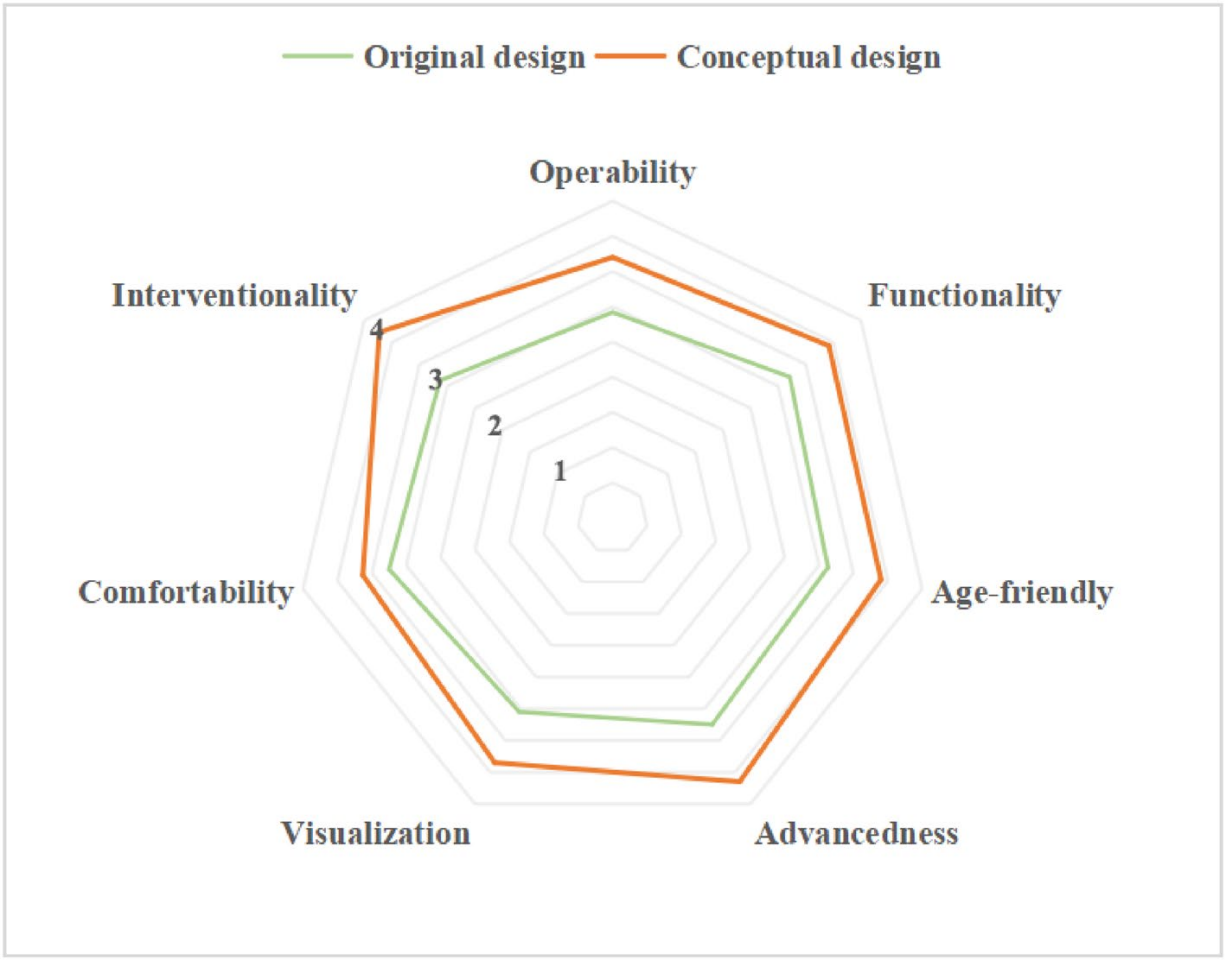
Radar diagram of the conceptual design of a cognitively friendly museum based on user needs.

A questionnaire survey in the form of a five-level scale is applied to the audience's satisfaction with the demand for cognitively friendly museum services. To ensure the scientific validity of the results of verifying the conceptual design of museum services, the research design evaluation is based on the mature scale of user experience, and the research scale is issued from the indexes of operability, functionality, age-friendly, advancedness, visualization, comfortability and interventionality, to carry out the test of the validity of the research program^19^. Through the use of the questionnaire star online research method, for the object of mild cognitive impairment dementia, their families, and carers. In order to be as scientific as possible, we conducted a 1:1 survey between regular users and targeted users when selecting candidates for questionnaire feedback on the design of older adults with dementia. A total of 300 questionnaires were sent out, and 292 valid questionnaires were recovered. The results show (Table 10, Fig. 7) that the average score of the original design sample attributes is 3.13, while the conceptual plan of museum service experience design based on user needs has an audience needs satisfaction value of 3.91. This shows that the cognitively friendly museum experience service system designed under the guidance of this study's methodology can improve audience satisfaction to a certain extent, which is instructive for the development and design of museum services.

**Table 10:** Changes in satisfaction of cognitively friendly museum service experience design.

Attribute	Original design	Conceptual design
Operability	2.92	3.70
Functionality	3.21	3.92
Age-friendly	3.13	3.90
Advancedness	3.25	4.15
Visualization	3.05	3.85
Comfortability	3.25	3.63
Interventionality	3.13	4.23

## Discussion

Cognitive impairment in older adults has always been the focus of attention in the global medical field. Currently, the application of providing cognitive intervening service experience for cognitively impaired dementia through museums has been slowly developed, and its application scenarios are getting richer and richer. Alzheimer's disease is a disease that is difficult to be intervened, so the design of the museum service experience is a complex process, which is worthy of further research by scholars. Cognitively friendly museum service design is a comprehensive systematic project, and this study focuses on the pre-conceptual design stage and does not involve the performance testing of the cognitively friendly museum experience in the later stage. This method provides a preliminary theoretical foundation for the development of cognitively friendly museum experience service design, provides the possibility of realizing scientific and precise design requirements for mining, and helps and promotes the optimization and upgrading of the museum experience at a later stage.

Our research results in comparing the existing design of museums and the conceptual design of museums based on users' needs show that the Kano-AHP method is a commonly used optimization design analysis method, which has an important helping role in the conceptual service design stage of museums. Compared with traditional museum design research methods, the application of the Kano-AHP method is more accurate in capturing special user needs. Compared with previous research, the Kano-AHP method has two main advantages: firstly, the Kano model can effectively help the design team to identify user needs, which helps to ensure that the design elements can satisfy user expectations and needs; secondly, the use of AHP hierarchical analytical model for evaluation and optimization helps to refine the analysis of each user's needs, which effectively improves the design of the museum service design. and services. As a whole, through the Kano-AHP methodology research for museum service development and design practice, from the perspective of user needs, it is possible to understand the functional service design needs of cognitively impaired people in museums, and based on this, propose a comprehensive approach to optimize the design.

Based on extensive research and scientific calculations, we have demonstrated that people with dementia and carers can derive health-related benefits from optimizing museums and that displaying museum virtual scenarios or old artifacts can be effective in increasing the subjective well-being of people suffering from various health conditions. This is not dissimilar to the findings of previous researchers, Johnson^46^ et al. used a quasi-experimental crossover design and a mixed design ANOVA showed a significant increase in well-being for object handling and art viewing for people with dementia and carers, and Schall^47^ et al. carried out a study on the ARTEMIS intervention, which showed that an art museum based art intervention was able to improve the (subjective well-being, mood and quality of life) in people with dementia.

However, this approach has some shortcomings in the study, and the application of the Kano-AHP method in the design of cognitively friendly museum experience services needs to take into account a variety of factors, such as the impact of smart technology techniques and the performance of display devices on museum services. There are complex interrelationships between these factors, which need to be considered comprehensively. In the later research, we will test the experiential nature of cognitively friendly museum services, study the technology, equipment, and use of contextual displays to further optimize the experience of the museum, and realize the development of the museum experience towards specialized, storytelling, and intelligent high-quality services, to improve the attention of the special populations as well as the humanized services.

## Conclusion

To analyze the design of museum services for people with cognitive disabilities, this study takes immersion theory as a guide and builds a museum service experience design strategy that integrates the KANO model and the Analysis of Hierarchy (AHP) method based on the needs of people with cognitive disabilities and obtains the perceptual elements affecting the design of cognitively friendly museums, which will be used as the principles of museum design. After a large number of research studies and interviews with scholars, the order of important attributes that should be considered in experience design is identified to avoid planners and designers from blindly carrying out the service experience design of museums. The study found that it can greatly improve social help for people with dementia, as well as improve the cognitive experience of people with dementia. It can comprehensively and effectively study the process of mutual synergistic development between museum services, aging people with cognitive disabilities, and social groups.

Despite these strengths, this study still has some shortcomings and limitations in terms of thoroughness, such as the lack of large sample size, but this does not affect the conclusions of the study. In future research, more consideration will be given to the stakeholders used in the study, further interviews with people with advanced dementia will be conducted to dig deeper into their actual needs, and the specific process of experience design for museum services will be examined in order to obtain more accurate data and theoretical knowledge, and to provide more valuable design suggestions for people with cognitive impairment in old age.

## Data Availability

The datasets generated and/or analysed during the current study are not publicly available due to privacy or ethical restrictions but are available from the corresponding author on reasonable request.

## References

[1] Sunjaya AP (2022). Uplifting primary care through the electronic health record. Ann. Fam. Med..

[2] Jia L, Du Y, Chu L, Zhang Z, Li F, Lyu D, Qiu Q (2020). Prevalence, risk factors, and management of dementia and mild cognitive impairment in adults aged 60 years or older in China: a cross-sectional study. Lancet Public Health.

[3] Zeisel, J., Bennett, K., & Fleming, R.World Alzheimer Report 2020: Design, dignity, dementia: Dementia-related design and the built environment (2020).

[4] Schelterns P, Feldman H (2003). Treatment of Alzheimer's disease; current status and new perspectives. Lancet Neurol..

[5] Jarrott SE, Kwack HR, Relf D (2002). An observational assessment of a dementia-specific horticultural therapy program. Hort. Technol..

[6] Yakimicki ML, Edwards NE, Richards E, Beck AM (2019). Animal-assisted intervention and dementia: A systematic review. Clin. Nurs. Res..

[7] Hu M, Zhang P, Leng M, Li C, Chen L (2018). Animal-assisted intervention for individuals with cognitive impairment: A meta-analysis of randomized controlled trials and quasi-randomized controlled trials. Psychiatry Res..

[8] Phillips LJ, Reid-Arndt SA, Pak Y (2010). Effects of a creative expression intervention on emotions, communication, and quality of life in persons with dementia. Nurs. Res..

[9] Filan SL, Llewellyn-Jones RH (2006). Animal-assisted therapy for dementia: A review of the literature. Int. Psychogeriatr..

[10] Serpell J, McCune S, Gee N, Griffin JA (2017). Current challenges to research on animal-assisted interventions. Appl. Dev. Sci..

[11] Chatterjee, H., & Noble, G. Museums, health and well-being. Routledge (2016).

[12] Dodd, J., & Jones, C. Mind, body, spirit: How museums impact health and wellbeing. University of Leicester (2014).

[13] Xiao W, Zhang N (2017). Whole-course management of Alzheimer's disease. Chin. J. Geriatr..

[14] Clare L, Marková IS, Roth I, Morris RG (2011). Awareness in Alzheimer's disease and associated dementias: Theoretical framework and clinical implications. Aging Mental Health.

[15] Xu W, Dai TT, Shen ZY, Yao YJ (2021). Effects of technology application on museum learning: A meta-analysis of 42 studies published between 2011 and 2021. Interact. Learn. Environ..

[16] King, B., & Lord, B. (Eds.). The manual of museum learning. Rowman & Littlefield (2015).

[17] Crooke, E. Museums and community: Ideas, issues and challenges. Routledge (2008).

[18] Amorim JSCD, Leite RC, Brizola R, Yonamine CY (2019). Virtual reality therapy for rehabilitation of balance in the elderly: A systematic review and META-analysis. Adv. Rheumatol..

[19] Schmidt, A. Context-aware computing: context-awareness, context-aware user interfaces, and implicit interaction. *Encyclop. Hum. Comput. Interact*, 2nd Ed (2013).

[20] Song JJ (2019). Virtual reality for vestibular rehabilitation. Clin. Exp. Otorhinolaryngol..

[21] Fritsch T, Kwak J, Grant S, Lang J, Montgomery RR, Basting AD (2009). Impact of TimeSlips, a creative expression intervention program, on nursing home residents with dementia and their caregivers. Gerontologist.

[22] Jeng MY, Pai FY, Yeh TM (2017). The virtual reality leisure activities experience on elderly people. Appl. Res. Qual. Life.

[23] Lin, R., Chen, H. Y., Li, H., & Li, J. Effects of creative expression therapy on Chinese elderly patients with dementia: an exploratory randomized controlled trial. Neuropsychiatr. Dis. Treatment 2019; 1: 2171–2180. 10.2147/NDT.S200045 PMC6679675 31440055

[24] Syed-Abdul S, Malwade S, Nursetyo AA, Sood M, Bhatia M, Barsasella D, Li YCJ (2019). Virtual reality among the elderly: a usefulness and acceptance study from Taiwan. BMC Geriatr..

[25] Epstein S (1994). Integration of the cognitive and the psychodynamic unconscious. Am. Psychol..

[26] Kim O, Pang Y, Kim JH (2019). The effectiveness of virtual reality for people with mild cognitive impairment or dementia: A meta-analysis. BMC psychiatry.

[27] Coyle H, Traynor V, Solowij N (2015). Computerized and virtual reality cognitive training for individuals at high risk of cognitive decline: Systematic review of the literature. Am. J. Geriatr. Psychiatry.

[28] Park MJ, Kim DJ, Lee U, Na EJ, Jeon HJ (2019). A literature overview of virtual reality (VR) in treatment of psychiatric disorders: Recent advances and limitations. Front. Psychiatry.

[29] Alamirah H, Schweiker M, Azar E (2022). Immersive virtual environments for occupant comfort and adaptive behavior research–A comprehensive review of tools and applications. Build. Environ..

[30] Jerald, J., & Marks, R. Human-centered design for VR interactions. In *ACM SIGGRAPH 2016 Courses*, 1–60 (2016).

[31] Biocca F, Delaney B (1995). Immersive virtual reality technology. Commun. Age Virtual Real..

[32] Perttula, A., Kiili, K., Lindstedt, A., & Tuomi, P. Flow experience in game based learning—a systematic literature review (2017).

[33] Satava, R. M., & Jones, S. Medical applications of virtual reality. In Virtual and adaptive environments, 325–343 (2003).

[34] Garcia-Betances RI, Jiménez-Mixco V, Arredondo MT, Cabrera-Umpiérrez MF (2015). Using virtual reality for cognitive training of the elderly. Am. J. Alzheimer's Dis. Other Dementias.

[35] Baus O, Bouchard S (2014). Moving from virtual reality exposure-based therapy to augmented reality exposure-based therapy: A review. Front. Hum. Neurosci..

[36] Flynn S, Palma P, Bender A (2007). Feasibility of using the Sony PlayStation 2 gaming platform for an individual poststroke: A case report. J. Neurol. Phys. Ther..

[37] Dalkey, N. C. Delphi: An introduction to technological forecasting. Routledge, pp. 25–30 (2018).

[38] Al-Harbi, Kamal M. Al-Subhi. "Application of the AHP in project management." *Int. J. Proj. Manag.* **19**(1), 19–27 (2001).

[39] Manera, V., Chapoulie, E., Bourgeois, J., Guerchouche, R., David, R., Ondrej, J., & Robert, P. A feasibility study with image-based rendered virtual reality in patients with mild cognitive impairment and dementia. *PloS one* **11**(3), e0151487 (2016). 10.1371/journal.pone.0151487 PMC4798753 26990298

[40] Tontini, G. Integrating the Kano model and QFD for designing new products. *Total Qual. Manag.* **18**(6), 599–612.

[41] Podvezko V (2007). Application of AHP technique. J. Bus. Econ. Manag..

[42] Mikulić J, Prebežac D (2011). A critical review of techniques for classifying quality attributes in the Kano model. Manag. Serv. Qual. Int. J..

[43] Langa KM, Levine DA (2014). The diagnosis and management of mild cognitive impairment: A clinical review. JAMA.

[44] The delphi method (1975). Reading.

[45] Rodriguez, R. Models, methods, concepts and applications of the analytic hierarchy process (Book Reviews). *Interfaces* **32**(6), 93–94 (2002).

[46] Johnson J, Culverwell A, Hulbert S, Robertson M, Camic PM (2017). Museum activities in dementia care: Using visual analog scales to measure subjective wellbeing. Dementia.

[47] Schall A, Tesky VA, Adams AK, Pantel J (2018). Art museum-based intervention to promote emotional well-being and improve quality of life in people with dementia: The ARTEMIS project. Dementia.

